# Prognostic differences among Grade Group 4 subgroups in robotic‐assisted radical prostatectomy

**DOI:** 10.1002/bco2.160

**Published:** 2022-06-02

**Authors:** Takeshi Sasaki, Shin Ebara, Tomoyuki Tatenuma, Yoshinori Ikehata, Akinori Nakayama, Daiki Kato, Masahiro Toide, Tatsuaki Yoneda, Kazushige Sakaguchi, Jun Teishima, Kazuhide Makiyama, Hiroshi Kitamura, Kazutaka Saito, Takuya Koie, Fumitaka Koga, Shinji Urakami, Takahiro Inoue

**Affiliations:** ^1^ Department of Nephro‐Urologic Surgery and Andrology Mie University Graduate School of Medicine Tsu Japan; ^2^ Department of Urology Hiroshima City Hiroshima Citizens Hospital Hiroshima Japan; ^3^ Department of Urology Yokohama City University Yokohama Japan; ^4^ Department of Urology University of Toyama Toyama Japan; ^5^ Department of Urology Dokkyo Medical University Saitama Medical Center Koshigaya Japan; ^6^ Department of Urology Gifu University Graduate School of Medicine Gifu Japan; ^7^ Department of Urology Tokyo Metropolitan Cancer and Infectious Diseases Center Komagome Hospital Tokyo Japan; ^8^ Department of Urology Seirei Hamamatsu General Hospital Hamamatsu Japan; ^9^ Department of Urology Toranomon Hospital Tokyo Japan; ^10^ Department of Urology Kobe City Medical Center West Hospital Kobe Japan

**Keywords:** Gleason score, grade group, prostate cancer, radical prostatectomy, robotic‐assisted

## Abstract

**Objectives:**

To investigate whether the International Society of Urological Pathology Grade Group 4 (GG 4) subgroups have different oncological outcomes in Japanese prostate cancer (PCa) patients undergoing robotic‐assisted radical prostatectomy (RARP).

**Patients and Methods:**

We conducted a retrospective multicentre cohort study in PCa patients undergoing RARP at 10 institutions in Japan. Pre‐ and post‐operative variables were collected from enrolled patients. We evaluated biochemical recurrence and clinical and pathological variables in the different GG 4 subgroups.

**Results:**

A total of 3195 patients were enrolled in the study. Among them, 298 patients with GG 4 tumours (pathological Gleason scores [GSs] of 3 + 5 [*N* = 37], 4 + 4 [*N* = 257] and 5 + 3 [*N* = 4]) based on RARP specimens were analysed. The median follow‐up period was 25.2 months. The 3‐year biochemical recurrence (BCR)‐free survival (BCRFS) rate in the overall population was 74.5%. The 3‐year BCRFS rates in the pathological GS 3 + 5, GS 4 + 4 and GS 5 + 3 subgroups were 93.8%, 71.9% and 50.0%, respectively (*P* = 0.01). In multivariate analysis, pathological GS based on RARP specimens, PSA levels at surgery, pathological T stage, pathological N stage and surgical margins were independent risk factors significantly associated with BCRFS. In particular, patients with pathological GSs 4 + 4 and 5 + 3 were at higher risk of BCR than patients with pathological GS 3 + 5 (hazard ratio 4.54, *P* = 0.03 and hazard ratio 11.2, *P* = 0.01, respectively). The study limitations include the lack of central pathological specimen evaluation.

**Conclusions:**

For patients with localized PCa undergoing RARP, pathological GS 4 + 4 and GS 5 + 3 were significantly associated with worse BCRFS than pathological GS 3 + 5. Pathological GS 3 + 5 may be overrated in GG 4. This observation emphasizes that primary and secondary GS should be considered to accurately stratify the risk of BCR after RARP.

## 1 INTRODUCTION

Several guidelines recommend radical prostatectomy (RP) for localized and some advanced prostate cancers (PCas).^1^ Robotic‐assisted RP (RARP) is suitable as a treatment modality for localized PCa. Although RARP is associated with less blood loss, a lower transfusion rate and a shorter hospitalization duration than open RP,^2^ there is no consensus as to whether RARP has better oncological outcomes than open RP.^2^, ^3^, ^4^ Approximately 2.5%–16% of PCa patients undergoing RARP still develop biochemical recurrence (BCR).^2^

The International Society of Urological Pathology (ISUP) developed the Gleason grading system for PCa in 2013.^5^ The five‐tier Grade Group (GG) system was accepted in the 2016 edition of the World Health Organization Classification of Tumours of the Urinary System and Male Genital Organs to be used in conjunction with Gleason score (GS).^6^ The five GGs include GG 1 (GS ≤ 6), GG 2 (GS 3 + 4), GG 3 (GS 4 + 3), GG 4 (GS 8) and GG 5 (GS 9–10). However, the definition of ISUP GG 4 is unsettled,^7^ as several studies have indicated a possible heterogeneity in GG 4. GG 4 includes all patients with GS 8 (i.e. GS 3 + 5, GS 4 + 4 and GS 5 + 3), regardless of their primary and secondary scores. A series of studies have evaluated associations of GS 8 (GS 3 + 5, GS 4 + 4 and GS 5 + 3) with clinicopathological variables and disease progression.^8^ Additionally, oncological outcomes have been evaluated among localized or metastatic PCa patients within GG 4 treated with RP, radiotherapy (RT) and androgen deprivation therapy (ADT).^8^ However, the impact of the GG 4 subgroups on oncological outcomes has never been investigated in Japanese PCa patients undergoing RARP. The ability to predict which patients are more likely to develop BCR after RARP could be helpful when choosing the best treatment strategy. Therefore, in this retrospective study, we investigated whether PCa patients undergoing RARP classified as having pathological GS 3 + 5, GS 4 + 4 and GS 5 + 3 had different clinicopathological variables and outcomes. This retrospective multicentre cohort study included PCa patients undergoing RARP at 10 institutions in Japan.

## 2 PATIENTS AND METHODS

### 2.1 Study participants

This study was approved by the Institutional Review Board of Mie University (approval number: H2021‐175) and the institutional review boards of all participating institutions. Patient consent was not required because of the retrospective nature of the study. The provisions of the ethics committee and ethics guidelines in Japan do not require written consent when the study information, such as existing documentation, is disclosed to the public for use in retrospective and/or observational studies.

We conducted a retrospective, multicentre cohort study in PCa patients undergoing RARP at 10 institutions (the MSUG94 group) in Japan between September 2012 and August 2021. The primary endpoint was overall treatment outcomes after RARP. The secondary endpoints were oncological outcomes (BCR, metastasis and development of castration resistance) and surgical complications of RARP. For preoperative staging, all patients underwent computed tomography (CT) of the chest, abdomen and pelvis; magnetic resonance imaging (MRI) of the pelvis; and bone scanning. After recurrence, all patients underwent CT of the chest, abdomen and pelvis and bone scanning. We excluded clinically metastatic PCa patients with cN1 and/or cM1. Preoperative information was gathered from patients, including patient age, height, weight, serum PSA level, clinical stage, biopsy GS, number and percentage of cancer‐positive biopsy cores, D'Amico risk stratification,^9^ Eastern Cooperative Oncology Group performance status, American Society of Anesthesiologists physical status and administration of neoadjuvant therapy before RARP. The pathological T and N stages of the surgical specimens were recorded, as were the pathological GS and the presence of extraprostatic extension, seminal vesicle invasion and positive surgical margins (PSMs). All tumours were staged according to the American Joint Committee on Cancer (AJCC) eighth edition cancer staging manual.^10^ All patients in the present study underwent RARP. The presence or absence of PLND, range of PLND and use of a nerve‐sparing approach were determined by the surgeon or the policy of each institution.

### 2.2 Pathological analysis

All prostatectomy specimens were sectioned according to the whole‐mount staining technique and evaluated according to the ISUP 2005 guidelines.^11^ The apex of the prostate was shaved perpendicular to the prostatic urethra. The bladder neck margin was coned from the specimen and sectioned perpendicularly. The remaining prostate tissue was completely sectioned at 3‐mm intervals along a plane perpendicular to the urethral axis.

### 2.3 Follow‐up schedule

Following surgery, all patients were assessed at 3‐month intervals according to their serum PSA levels. The date of BCR or PSA failure was defined as the date when the serum PSA level exceeded 0.2 ng/mL. If the PSA level did not decrease below 0.2 ng/mL after RARP, the date of BCR was defined as the date of RARP. Times to events were calculated from the day of surgery. Imaging for metastatic disease was left to the physician's judgement based on PSA levels and/or symptoms of recurrent disease. After biochemical progression, salvage RT, hormonal therapy and chemotherapy were performed. Castration‐resistant PCa (CRPC) was defined as either progressively rising PSA (two 50% increases over the nadir with a PSA > 2.0 ng/mL), despite a castration level (<50 ng/dL) of testosterone, as previously described.^12^

### 2.4 Statistical analysis

Associations of GS with categorical variables were assessed using the chi‐square test, and differences in continuous variables were analysed using the Kruskal‐Wallis test. BCR‐free survival (BCRFS) after RARP was analysed using the Kaplan–Meier method, and differences between groups were assessed by log‐rank test. Multivariate analysis was performed using the Cox proportional hazards regression model. Statistical analysis was performed using SPSS software, Version 22 (IBM Corporation, Armonk, NY, USA), and *P* < 0.05 was considered to be statistically significant.

## 3 RESULTS

### 3.1 Patient characteristics

A total of 3195 patients were enrolled in the study. Patients who received neoadjuvant therapy and those without clearly evaluated clinical and pathological findings were excluded. Finally, 298 patients with pathological GS 3 + 5 (*N* = 37, 12%), GS 4 + 4 (*N* = 257, 86%) and GS 5 + 3 (*N* = 4, 2%) with GG 4 tumours were analysed for oncological outcomes. Demographic data of the patients are presented in Table 1. The median age at surgery was 69 years. Overall, 108 (36%) patients had PSMs, and 172 (58%), 73 (24%), 53 (18%) and 19 (6%) had pathological T2 (pT2), pT3a, pT3b and pN1 disease, respectively (Table 1). When patients were stratified according to pathological Gleason patterns based on RARP specimens, a significant difference was observed with regard to the biopsy GS (*P* = 0.008) (Table 1). The numbers of median (range) nodes of pelvic lymphadenectomy in pathological GS 3 + 5, GS 4 + 4 and GS 5 + 3 were 12 (2–45), 6 (0–44) and 18 (12–20), respectively (*P* < 0.001) (Table 1).

**TABLE 1:** Patient characteristics

	ALL	GS 3 + 5	GS 4 + 4	GS 5 + 3	*P‐*value
Number of patients (%)	298 (100)	37 (12)	257 (86)	4 (2)	
Median follow‐up time (range) months	25.2 (0.3–104)	25.5 (1.9–71)	24.6 (0.3–104)	61.4 (40–88)	
Median age (range) at surgery years	69 (43–81)	68 (51–80)	70 (43–81)	69 (63–73)	0.20
Median PSA levels (range) at surgery ng/mL	8.7 (1.4–78)	9.1 (4–26)	8.6 (1.4–78)	8.6 (5.6–11)	0.80
Biopsy GS (%)					
6	22 (7)	1 (2)	21 (8)	0 (0)	0.008
7	92 (31)	21 (58)	70 (27)	1 (25)	
≥8	184 (62)	15 (40)	166 (65)	3 (75)	
Clinical stage at diagnosis (%)					
cT1	39 (13)	5 (13)	34 (13)	0 (0)	0.52
cT2	218 (73.5)	30 (82)	185 (72)	3 (75)	
cT3	40 (13)	2 (5)	37 (14.5)	1 (25)	
cTx	1 (0.5)	0 (0)	1 (0.5)	0 (0)	
Pathological T stage (%)					
pT2	172 (58)	22 (60)	148 (58)	2 (50)	0.47
pT3a	73 (24)	11 (30)	60 (23)	2 (50)	
pT3b	53 (18)	4 (10)	49 (19)	0 (0)	
Pathological N stage (%)					
pN0	217 (73)	28 (76)	187 (73)	2 (50)	0.16
pN1	19 (6)	0 (0)	18 (7)	1 (25)	
pNx (cN0)	62 (21)	9 (24)	52 (20)	1 (25)	
Median number of resected lymph nodes (range)					
[GS 3 + 5 (*N* = 28), GS 4 + 4 (*N* = 205), GS 5 + 3 (*N* = 3)]	7 (0–45)	12 (2–45)	6 (0–44)	18 (12–20)	<0.001
Surgical margins (%)					
Negative	188 (63)	23 (63)	162 (63)	3 (75)	0.44
Positive	108 (36)	13 (35)	94 (36.5)	1 (25)	
NA	2 (1)	1 (2)	1 (0.5)	0 (0)	
Nerve sparing (%)					
None	231 (78)	22 (60)	205 (80)	4 (100)	0.03
Unilateral	58 (19)	12 (32)	46 (18)	0 (0)	
Bilateral	9 (3)	3 (8)	6 (2)	0 (0)	

Abbreviations: GS, Gleason score; NA, not available; PSA, prostate‐specific antigen.

**FIGURE 1:**
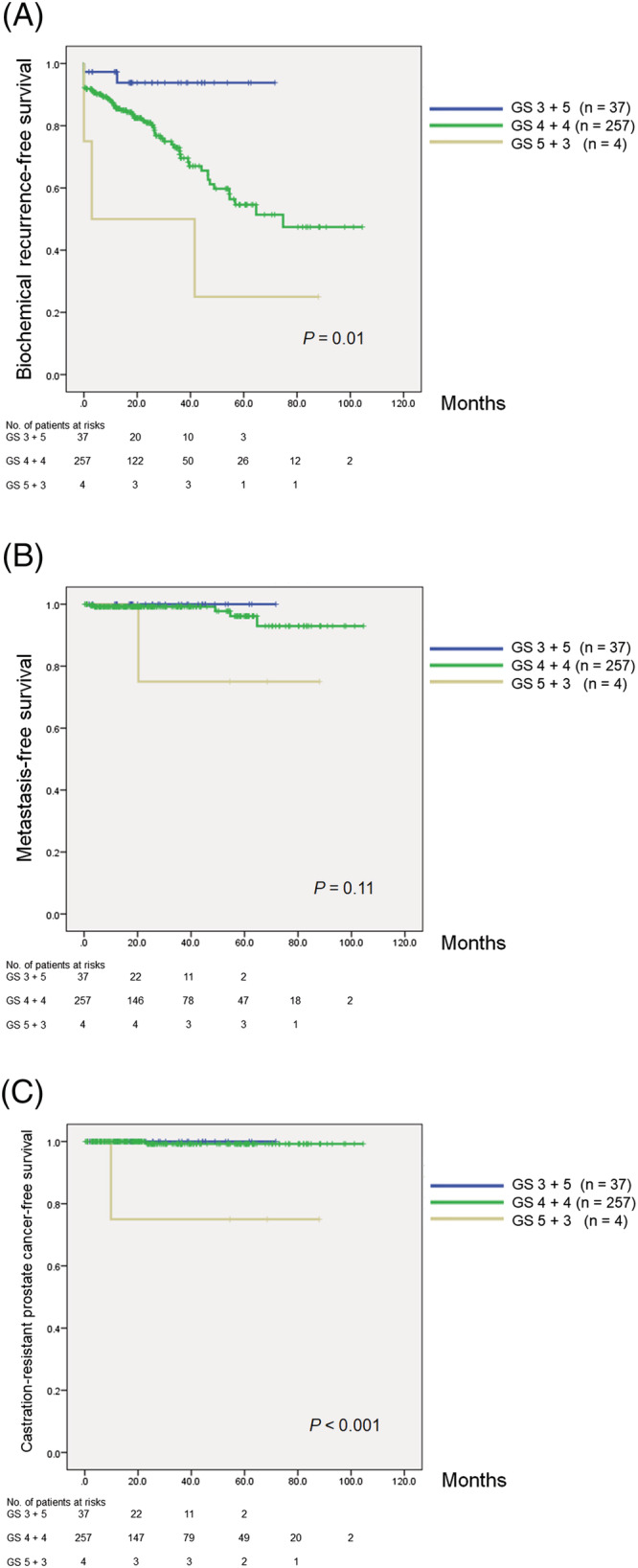
Kaplan–Meier estimates of biochemical recurrence‐free survival (BCRFS) (A), metastasis‐free survival (B) and castration‐resistant prostate cancer (CRPC)‐free survival (C) in patients who underwent robotic‐assisted radical prostatectomy. The 3‐year BCRFS rates were 93.8%, 71.9% and 50.0% for those with pathological GS 3 + 5, GS 4 + 4 and GS 5 + 3 tumours, respectively (A; *P* = 0.01). The 3‐year metastasis‐free survival rates were 100.0%, 99.2% and 75.0% for those with pathological GS 3 + 5, GS 4 + 4 and GS 5 + 3 tumours, respectively (B; *P* = 0.11). The 3‐year CRPC‐free survival rates were 100.0%, 99.3% and 75.0% for those with pathological GS 3 + 5, GS 4 + 4 and GS 5 + 3 tumours, respectively (C; *P* < 0.001). GS, Gleason score

The median follow‐up was 25.2 months, and 71 patients (23.8%) experienced BCR. The 3‐year and 5‐year BCRFS rates in the overall population were 74.5% and 57.8%, respectively. When patients were stratified according to pathological Gleason pattern, the 3‐year BCRFS rates were 93.8%, 71.9% and 50.0% for those with pathological GS 3 + 5, GS 4 + 4 and GS 5 + 3 tumours, respectively (Figure 1A; *P* = 0.01). The 3‐year metastasis‐free survival rates were 100.0%, 99.2% and 75.0% for those with pathological GS 3 + 5, GS 4 + 4 and GS 5 + 3 tumours, respectively (Figure 1B; *P* = 0.11). The corresponding 3‐year CRPC‐free survival rates were 100.0%, 99.3% and 75.0%, respectively (Figure 1C; *P* < 0.001).

In the univariate analysis, pathological GS based on RARP specimens, PSA levels at surgery, pT stage, pN stage, surgical margins and nerve‐sparing procedures were associated with BCRFS (Table 2). In the multivariate analysis, pathological GS based on RARP specimens, PSA levels at surgery, pT stage, pN stage and surgical margins were independent risk factors significantly associated with BCRFS (Table 2). In particular, patients with pathological GS 4 + 4 and GS 5 + 3 tumours were at a higher risk of BCR than patients with pathological GS 3 + 5 tumours (hazard ratio 4.54, *P* = 0.03 and hazard ratio 11.2, *P* = 0.01, respectively).

**TABLE 2:** Univariate and multivariate analysis assessing the role of pathological Grade Group 4 subgroups on the risk of biochemical recurrence (BCR) in 298 prostate cancer (PCa) patients based on robotic‐assisted radical prostatectomy specimens

Factors	Univariate	Multivariate
	Hazard ratio [95%CI] (*P*‐value)	Hazard ratio [95%CI] (*P*‐value)
Gleason score based on RARP specimens		
3 + 5	1 (Ref.)	1 (Ref.)
4 + 4	5.07 [1.24–20.7] (*P* = 0.02)	4.54 [1.10–18.7] (*P* = 0.03)
5 + 3	11.9 [1.98–72.0] (*P* = 0.007)	11.2 [1.78–71.2] (*P* = 0.01)
Age (years) at surgery	1.02 [0.98–1.06] (*P* = 0.26)	―
PSA levels (ng/ml) at surgery	1.04 [1.03–1.06] (*P* < 0.001)	1.02 [1.01–1.04] (*P* < 0.001)
Pathological T stage		
pT2	1 (Ref.)	1 (Ref.)
pT3a	3.61 [2.02–6.45] (*P* < 0.001)	2.73 [1.49–4.99] (*P* < 0.001)
pT3b	4.39 [2.43–7.93] (*P* < 0.001)	2.39 [1.18–4.83] (*P* = 0.01)
Pathological N stage		
pN0	1 (Ref.)	1 (Ref.)
pN1	6.11 [3.31–11.2] (*P* < 0.001)	3.27 [1.51–7.06] (*P* = 0.003)
pNx (cN0)	0.78 [0.38–1.59] (*P* = 0.50)	0.81 [0.38–1.73] (*P* = 0.60)
Surgical margins		
Negative	1 (Ref.)	1 (Ref.)
Positive	2.56 [1.59–4.11] (*P* < 0.001)	2.07 [1.24–3.48] (*P* = 0.005)
Nerve sparing		
None	1 (Ref.)	
Unilateral or bilateral	0.47 [0.24–0.90] (*P* = 0.02)	―

Abbreviation: RARP, robotic‐assisted radical prostatectomy.

## 4 DISCUSSION

In our study, we evaluated the prognostic differences among PCa patients undergoing RARP with pathological GS 3 + 5, GS 4 + 4 and GS 5 + 3 tumours. Previous studies from Western countries demonstrated that PCa patients undergoing RP with pathological GS 3 + 5 tumours had significantly better outcomes than those with GS 4 + 4 tumours.^13^, ^14^, ^15^, ^16^, ^17^ In our large, multicentre, national study of patients who received RARP, we confirmed the heterogeneity of pathological GG 4 in predicting BCR. We demonstrated that patients undergoing RARP with pathological GS 4 + 4 and GS 5 + 3 tumours were at higher risk of BCR than those with pathological GS 3 + 5 tumours. This observation emphasizes that primary and secondary GSs should be considered to accurately stratify the risk of BCR after RARP.

Epstein et al. reported that the vast majority of patients in GG 4 had pathological GS 4 + 4 tumours; therefore, they did not consider it useful to further separate patients with pathological GS 3 + 5 or GS 5 + 3 tumours based on the frequencies alone.^18^, ^19^ In their study investigating the incidences of GS 3 + 5 and GS 5 + 3 tumours^19^ in 20 824 RP cases graded by uropathology experts, only 39 (0.2%) had 3 + 5 tumours, and 4 (0.02%) had 5 + 3 tumours. In our cohort, only four patients (0.1%) had GS 5 + 3 tumours (Table 1). Therefore, GS 5 + 3 tumours were extremely rare in our cohort. However, of 3195 patients, 37 (1.1%) had GS 3 + 5 tumours, indicating it may be less rare in some cohorts. The incidences of GS 3 + 5 and GS 5 + 3 tumours within RP specimens in real‐world data are similar to or much higher than those in our cohort.^14^, ^16^, ^17^

The Epstein group also proposed modifying GGs by incorporating a prognostic grade grouping that accurately reflects prognosis.^5^, ^18^ They showed that the 5‐year BCRFS rate for GG 4 based on RP pathology was 48%.^18^ The 5‐year BCRFS rate for GG 4 in our study was similar (57.8%). In 2016, the AJCC eighth edition established criteria to evaluate prediction models, including clinical T stage, pathological T stage, N stage, M stage, PSA titres and GG.^10^ In this model, GG 4 included GS 4 + 4, GS 3 + 5 and GS 5 + 3. However, it is unclear whether GS 3 + 5 and GS 5 + 3 have equivalent outcomes to GS 4 + 4. Recently, large international studies developed points‐based GS staging systems for predicting BCR and cancer‐specific mortality in patients with non‐metastatic PCa.^20^, ^21^ Interestingly, in these studies, a primary pattern of 5 (GS 5 + 3) was evaluated separately from other primary patterns (GS 4 + 4 or GS 3 + 5).^20^, ^21^ To address this, several studies have attempted to evaluate the associations of GG 4 subgroups with clinicopathological variables and disease progression.

Table 3 summarizes several lines of clinical evidence showing the prognostic impact of different GG 4 subgroups (GS 3 + 5 vs. GS 4 + 4 vs. GS 5 + 3) on PCa outcomes.^13^, ^14^, ^15^, ^16^, ^17^, ^22^, ^23^, ^24^, ^25^, ^26^, ^27^ Rushoven et al. demonstrated that patients with biopsy Gleason pattern 5 tumours (GS 3 + 5 or GS 5 + 3) had worse survival than those with biopsy GS 4 + 4 tumours among patients with metastatic or localized PCa undergoing RT.^22^, ^23^ Because these studies focused on biopsy Gleason pattern 5, GS 3 + 5 and GS 5 + 3 tumours were included in the same category. Similarly, Huynh et al. and Lu et al. compared men with GS 3 + 5/5 + 3 tumours and those with GS 4 + 4 tumours based on the biopsy GSs.^26^, ^27^ Huynh et al. demonstrated that PCa‐specific mortality (PCSM) and all‐cause mortality were significantly higher among men undergoing RT and ADT with GS 3 + 5/5 + 3 tumours than among those with GS 4 + 4 tumours,^26^ but Lu et al. found no significant differences in PCSM risk between men undergoing RP, RT, ADT and chemotherapy with biopsy GS 3 + 5/5 + 3 tumours and those with GS 4 + 4 tumours. However, the authors did not separate GS 3 + 5 and GS 5 + 3 tumours.

**TABLE 3:** Summary of the prognostic impact of different Gleason score 8 (3 + 5 vs. 4 + 4 vs. 5 + 3) on PCa outcome

Study	Region	Patients and treatment	GS specimens	Outcome	Follow‐up (months)
Rusthoven et al. 2014^23^	USA	GS 3 + 5 and 5 + 3: *N* = 167, GS 4 + 4: *N* = 906, ALL	Biopsy	Gleason pattern 5 (3 + 5 or 5 + 3) was associated with inferior survival when compared with 4 + 4 disease	48
Rusthoven et al. 2015^24^	USA	GS 3 + 5 and 5 + 3: N = 359, GS 4 + 4: N = 2545, RT	Biopsy	Gleason pattern 5 (3 + 5 or 5 + 3) was associated with inferior survival when compared to 4 + 4 disease.	72
Mahal et al. 2016^25^	USA	GS 3 + 5: *N* = 2668, GS 4 + 4: *N* = 21 503, GS 5 + 3: *N* = 892, ALL	Biopsy/RP	PCSM of GS 3 + 5 and GS 4 + 4 are similar, but GS 5 + 3 is high risk of PCSM	36
Van den Bergh et al. 2016^13^	International	GS 3 + 5: N = 62, GS 4 + 4: *N* = 134, GS 5 + 3: *N* = 15, RP	Biopsy/RP	BCR rate of GS 3 + 5 was significantly favourable than GS 4 + 4	20.4
Harding‐Jackson et al. 2016^20^	USA	GS 3 + 5: *N* = 58, GS 4 + 4: *N* = 121, GS 5 + 3: *N* = 0, ALL	Biopsy	GS 4 + 4 and GS 3 + 5 have a similar prognosis	33.4
Huynh et al. 2016^26^	USA	GS 3 + 5 and 5 + 3: *N* = 41, GS 4 + 4: *N* = 421, RT + ADT	Biopsy	PCSM and ACM were higher for men with GS3 + 5/5 + 3 than for men with GS 4 + 4	91.2
Gandaglia et al. 2017^14^	International	GS 3 + 5: *N* = 295, GS 4 + 4: *N* = 651, GS 5 + 3: *N* = 143, RP	RP	Men with GS 3 + 5 are at reduced risk of recurrence compared with men with primary GS 4 or 5	83
Lu et al. 2018^27^	Australia	GS 3 + 5: *N* = 55, GS 4 + 4: *N* = 664, GS 5 + 3: *N* = 21, ALL	Biopsy	No significant difference in PCSM risk between GS 4 + 4 and GS 3 + 5/5 + 3	60
Mori et al. 2021^15^	International	GS 3 + 5: *N* = 190, GS 4 + 4: *N* = 1557, GS 5 + 3: *N* = 44, RP	Biopsy	GS 4 + 4 was significantly associated with worse BCRFS than GS 3 + 5	75
Mori et al. 2021^16^	International	GS 3 + 5: *N* = 189, GS 4 + 4: *N* = 500, GS 5 + 3: *N* = 98, RP	RP	GS 4 + 4 was significantly associated with worse BCRFS than GS 3 + 5	86
Hollemans et al. 2021^17^	Netherlands	GS 3 + 5: *N* = 76, GS 4 + 4: *N* = 46, GS 5 + 3: *N* = 18, RP	RP	BCR and metastases occur more often in GS 4 + 4 than GS 3 + 5/5 + 3	68.7
Present study	Japan	GS 3 + 5: *N* = 37, GS 4 + 4: *N* = 257, GS 5 + 3: *N* = 4, RARP	RP	GS 4 + 4 and GS 5 + 3 were significantly associated with worse BCRFS than GS 3 + 5	25.2

Abbreviations: ACM, all‐cause mortality; ADT, androgen deprivation therapy; BCRFS, biochemical recurrence‐free survival; GS, Gleason score; PCSM, prostate cancer‐specific mortality; RARP, robotic‐assisted radical prostatectomy. RP, radical prostatectomy; RT, radiotherapy.

Conversely, several recent studies evaluated GS 3 + 5 and GS 5 + 3 separately.^13^, ^14^, ^15^, ^16^, ^24^, ^25^ Two studies including PCa patients undergoing multimodality treatment concluded that the PCSM and OS of patients with GS 3 + 5 and GS 4 + 4 disease are similar,^24^, ^25^ but patients with GS 5 + 3 disease have a higher risk of PCSM.^24^ However, these studies have varied initial therapeutic approaches. In our study, although the follow‐up period was relatively short, we included only patients undergoing RARP. We found that the metastasis‐free survivals and CRPC‐free survivals of patients with GS 3 + 5 and GS 4 + 4 disease were similar, but patients with GS 5 + 3 disease had higher risks of metastasis and CRPC (Figure 1B,C). Further follow‐up will provide a more definitive conclusion.

Intriguingly, multiple studies examining BCR after RP revealed that the BCR rate of patients with pathological GS 4 + 4 tumours was worse than that of patients with GS 3 + 5 tumours.^13^, ^14^, ^15^, ^16^, ^17^ Our results also indicated that patients with pathological GS 4 + 4 tumours undergoing RARP were also at higher risk of BCR than those with GS 3 + 5 tumours. Moreover, in our multivariate analysis for BCRFS after RARP, pathological GS 3 + 5, lower PSA, pT2, pN0 and negative surgical margins were significant favourable prognostic factors, with GS being the strongest prognostic factor (Table 2). Gandaglia et al. demonstrated that pathological GS 4 + 4 was associated with a 1.38‐fold higher risk of recurrence than pathological GS 3 + 5,^14^ and Mori et al. also demonstrated that pathological GS 4 + 4 was associated with a 1.81‐fold higher risk of recurrence than pathological GS 3 + 5 in multivariate analysis for BCRFS after RP.^16^ Hollemans et al. revealed that invasive cribriform and/or intraductal carcinoma was observed more frequently in RP specimens of GS 4 + 4 tumours (93%) than in those of GS 3 + 5 tumours (47%; *P* < 0.001).^17^

The present study has several limitations. First, this was a retrospective, multicentre cohort study and therefore has an inherent potential for bias. Second, we acknowledge the lack of a centralized pathological review involving biopsy and pathological GSs. GS 8 is known to be a very heterogeneous disease including highly variable quantities of Gleason 3, 4 and 5 growth patterns, which may lead to significant inter‐observer variability in tumour grading.^17^ Third, we had few cases of GS 5 + 3 in our cohort. Last, the follow‐up period was relatively short; therefore, it may be insufficient to precisely identify the predictive factors of BCR, and there is a lack of cancer‐specific survival data after RARP. However, we believe that further prospective studies with large cohort sizes will enable identification of critical roles of the heterogeneous GG 4 in predicting outcomes.

## 5 CONCLUSIONS

In conclusion, GS 4 + 4 and GS 5 + 3 in patients with localized PCa treated by RARP were associated with significantly worse BCRFS than GS 3 + 5. Pathological GS 3 + 5 may be overrated in GG 4. This observation emphasizes that primary and secondary GS should be considered to accurately stratify the risk of BCR after RARP. This could be useful for selecting the best treatment strategy by predicting which patients are more likely to develop BCR after RARP.

## CONFLICT OF INTEREST

The authors declare no conflict of interest.

## ETHICS STATEMENT

The protocol for this research project has been approved by a suitably constituted Ethics Committee of each institution, and it conforms to the provisions of the Declaration of Helsinki (Mie University Hospital Clinical Research Ethics Committee, Approval No. H2021‐175 and institutional review boards). As the study design was retrospective and observational, the requirement for obtaining informed consent from the participants was waived.

## AUTHOR CONTRIBUTIONS

Takeshi Sasaki: Data collection and management, data analysis, manuscript writing/editing. Shin Ebara: Protocol/project development, data collection and management. Tomoyuki Tatenuma: Data collection and management. Yoshinori Ikehata: Data collection and management. Akinori Nakayama: Data collection and management. Daiki Kato: Protocol/project development, data collection and management. Masahiro Toide: Data collection and management. Tatsuaki Yoneda: Protocol/project development, data collection and management. Kazushige Sakaguchi: Data collection and management. Jun Teishima: Protocol/project development and supervision. Kazuhide Makiyama: Protocol/project development and supervision. Hiroshi Kitamura: Protocol/project development and supervision. Kazutaka Saito: Protocol/project development and supervision. Takuya Koie: Protocol/project development and supervision. Fumitaka Koga: Protocol/project development and supervision. Shinji Urakami: Protocol/project development and supervision. Takahiro Inoue: Protocol/project development, data management, manuscript writing/editing.
